# A Case of Blau Syndrome

**DOI:** 10.1155/2014/216056

**Published:** 2014-05-04

**Authors:** Krati Chauhan, Clement Michet

**Affiliations:** Division of Rheumatology, Department of Internal Medicine, Mayo Clinic, Rochester, MN 55901, USA

## Abstract

We present a case of systemic granulomatous disorder/Blau syndrome. A patient was seen at our clinic with a diagnosis of Juvenile Idiopathic Arthritis (JIA). He was diagnosed with polyarticular JIA when he was two years old, at that time primary manifestations included inflammation of the hand and wrist joints bilaterally, later he developed ocular symptoms, which were attributed to JIA. He had liver, skin, pulmonary manifestations, and diagnostic workup including biopsy revealed granulomatous inflammation of these sites. During the diagnostic workup, he had worsening of ocular complaints, retinal exam showed panuveitis with multifocal choroiditis. These ocular findings are not seen in JIA, this, along with his other systemic manifestations, led us to revisit the diagnosis. Laboratory testing for genetic mutation for Blau syndrome was done and came back positive. Now all of his systemic findings were placed under one umbrella of systemic granulomatous syndrome/Blau syndrome. Due to worsening of ocular manifestations, he was started on Adalimumab with marked improvement of ocular and systemic manifestations and is followed by team that consists of Rheumatologist, Ophthalmologist, and Gastroenterologist.

## 1. Case

Our patient is a 23-year-old gentleman who was referred to the rheumatology clinic for continual management of Juvenile Idiopathic Arthritis (JIA). He was diagnosed with polyarticular JIA when he was two years old. His presentation in the beginning consisted of symmetric arthritis involving fingers and wrists. As a consequence of arthritis, he underwent bilateral wrist fusion, which was done prior to him being seen at our clinic, hence we do not have the results of synovial biopsy. His initial treatment consisted of nonsteroidal anti-inflammatory (NSAIDs), later on methotrexate was added. He remained on methotrexate for about ten years; it was discontinued when the patient was diagnosed with lung problems, primarily bronchitis, the symptoms improved with increasing the dose of prednisone. Subsequently, he was started on azathioprine, remained on it for three years, but was discontinued due to liver function abnormalities. After this, he was started on hydroxychloroquine. About ten years ago, the patient was diagnosed with iritis, treated with prednisone, which was tapered off to a dose of 3 mg once a day. There was no family history of arthritis or other autoimmune conditions.

At the present clinic visit, he denied joint pain, swelling, redness, and morning stiffness, and had no eye complaints. The rest of the review of the system was also negative. On joint examination he had flexion contracture of fingers of both hands and bilateral wrist fusion. There was no active synovitis, and remainder of the systemic examination was unremarkable. Labs including completer blood count, blood chemistry, and markers of inflammation were within normal limits. In summary, a 23-year-old gentleman with polyarticular JIA primarily with joint and ocular manifestations, pulmonary and liver abnormalities attributed to drug exposure. His disease was considered stable, and it was recommended to continue hydroxychloroquine and prednisone, along with follow-up Rheumatology clinic visits.

He was also seen by gastroenterology for elevated liver enzymes, which had never normalized (Alkaline Phosphate: 716:  (45–115), AST: 71 (8–48), ALT: 80 (7–55)). Patient denied nausea, vomiting, abdominal pain, itching, fever, and skin discoloration and physical examination did not show any skin findings of chronic liver disease, and the edge of the liver was palpable at the rib margin and was firm in texture. Autoimmune markers: anti-smooth muscle antibodies, anti-mitochondrial antibody, and hepatitis serologies for hepatitis B and C were negative. An MRI of liver which showed nodularity and fibrosis, and MR elastography showed modest elevation of liver stiffness: 4.5 (normal: 0–2.9). A liver biopsy, showed granulomatous hepatitis with periportal and early septal fibrosis. He was started on ursodiol, as his picture was primarily cholestatic and ursodiol has been shown to be beneficial in granulomatous bile duct disease. After treatment with ursodiol, there was some improvement in alkaline phosphatase (568, 538) and AST and ALT remained mildly elevated as before.

Since his initial pulmonary involvement, he was followed up by his pulmonologist lung examination was unremarkable, pulmonary function test was read as FVC: 3.14 liters (63% of predicted), FEV1: 1.72 liters (41%) of predicted, uncorrected DLCO 20.61 (57% of predicted). Chest CT scan was done which showed micronodules throughout bilateral lung fields consistent with the small airway disease: bronchiolitis obliterans. Transbronchial lung biopsy showed granulomatous inflammation. These findings along with improvement of his pulmonary symptoms with steroids were attributed to existing rheumatologic diagnosis of arthritis. He also developed erythematous lesion of scalp, and skin biopsy was positive for granulomatous inflammation.

In the interim, he developed blurry vision and was referred to be evaluated by retina specialist. Examination by retina specialist showed multifocal choroiditis with panuveitis. Findings of multifocal choroiditis is very atypical of juvenile idiopathic arthritis, this led to a discussion between ophthalmologist and rheumatologist. His other systemic manifestations were also put together, which had previously showed granulomatous inflammation of liver, lungs, and skin. At this point, consideration was given to whether he has systemic granulomatous disease. DNA PCR and DNA sequencing for all twelve coding regions of NOD2 was done and it showed heterozygous mutation in Glu498Gly. Patient's family members are still considering genetic testing, as none of them are symptomatic, they have deferred it at present. He does not have children. Since we do not have information about mutation present in parents, hence, it is difficult to say whether the mutation is inherited or sporadic in nature.

With the above clinical presentation and gene mutation, patient was diagnosed with systemic granulomatous inflammation: Blau syndrome and he was started on Adalimumab. With the initiation of Adalimumab, there was marked improvement in Ophthalmologic symptoms. Alkaline phosphate continues to improve, (337, 259, 215), AST (49), and ALT (55) have also improved, though there has been fluctuations in AST and ALT with some values higher than the previous ones. MR elastography shows improvement in liver stiffness (3.5, 3.2) which is very close to the normal value of 2.9, and he has not developed any cholestatic symptoms. Repeated CT scans of chest have remained stable, with presence of micronodules. Pulmonary function tests are similar to before, consistent with obstructive pulmonary process (FVC: 3.11, 67% of predicted, FEV1: 1.81, 46% of predicted, FEV1/FVC 58, 60% of predicted, DLCO: 34.8, 54% of predicted), and there has been no reoccurrence of the scalp lesions.

## 2. Discussion

Blau syndrome is a rare granulomatous autoinflammatory syndrome [1], first described by pediatrician Blau in the year 1985 as a dominantly inherited disorder [2]. Miceli-Richard et al. identified the responsible gene, the caspase recruitment gene: NOD2, mapped to chromosomal region 16q12.1-13 in 2001 [3], it has 12 exons spanning 36 kilobase. Ten different mRNA, all capable of coding for protein, have been identified, [4].

Initially, three missense mutations (R334Q, R334W, and L469F) in nucleotide binding domain of NOD2 gene in four French and German families [3] were discovered. Till date, ten Blau associated genetic mutations have been identified in the NOD2 gene, mostly in heterozygous state. Two of the mutations (R334Q and R334W) are responsible for 50% of mutated alleles [5]. An amino acid substitution of Arginine to tryptophan at position 334 (R334Q) was reported in two of the initially described families [3] and the majority of described mutations involves this evolutionary conserved region. However, mutations in other locations have been described and new mutations continued to be added to the already reported ones. Mutation reported in our case Glu498Gly has not been reported previously, and represents a new mutation.

NOD2 encodes a multidomain protein consisting of 1040 amino acids, which is a member of NOD-like receptor family (NLR). It is an integral part of the innate immunity, and is expressed in macrophages, dendritic cells, and epithelial cells [4]. NOD-like receptors are present in the cytoplasm and recognize intracellular molecules, [6].

NOD2 has a characteristic tripartite structure. The carboxyl terminal region is the sensory region, consists of leucine rich repeat region (LRR); it mediates protein-protein interactions and pattern recognition of various pathogens and host molecules, [6, 7]. Central domain is the nucleotide binding and oligomerization (NACHT) domain which is responsible for oligomerization, formation of high molecular weight complexes, and subsequent activation of NOD2. Nitrogen terminal is the effector region termed as the, caspase recruitment domain (CARD). This region mediates signal transduction to downstream target, leading to activation of nuclear factor kappa b (NF-*κ*B). NOD2 is the member of the NLRC subgroup of the NLR and consists of 6 LRR, 1 NACHT, and 2 CARD domains [4].

NOD2 is present in the cytoplasm in an inactivated state. Upon binding to a ligand at the carboxyl terminal, oligomerization through NACHT domain occurs. This oligomerization exposes the CARD in the effector nitrogen terminal, leading to activation of NOD2. CARD of activated NOD2 interacts with serine-threonine kinase RICK or Receptor Interacting Protein-2 (RIP-2). RICK ubiquinates IkB kinase (IKK), transforming IKK to an active state. Activated IKK phosphorylates IkB, relieving inhibition of Nuclear factor kappa b (NF-*κ*B) [4]. Activated NF-*κ*B translocates to nucleus, resulting in transcription of inflammatory genes [8]. In Blau Syndrome, there is gain of function mutation of NOD2 gene with increase in baseline NF-*κ*B activity, resulting in increased transcription of inflammatory genes, which culminates into the multisystem granulomatous inflammation [4].

Individuals with Blau syndrome generally present in early childhood [4]. Articular, skin, and ocular involvement is considered characteristic. Arthritis is the most common presentation, usually appears in the first decade of life, as seen in our patient [9]. Wrist, metacarpophalangeal, metatarsophalangeal, proximal interphalangeal, ankles, and elbows are the most common joints involved [10]. Hypertrophic tenosynovitis and synovial cyst results in Camptodactyly [4] and chronic arthritis leads to finger deformity and ankylosis of wrist joint [11].

Skin manifestation usually begins as maculopapular erythematous rash, which evolves into multiple papules, later on becoming confluent. Appearance is often symmetric on extremities and trunk. The presentation is markedly varied, as our patient presented with erythematous lesions of scalp, and in some cases lesions heal, leaving pitted scars, [12].

The most significant morbidity of Blau syndrome is ocular involvement [2, 13]. Recurrent anterior uveitis is frequent, characterized by ocular pain, blurry vision, and photophobia. It can evolve into panuveitis, with multifocal choroiditis, usually bilateral. Our patient initially developed iritis which was managed with corticosteroids, subsequently after many years he again complained of blurry vision and was found to have multifocal choroiditis with panuveitis. These ocular findings in the context of rest of the systemic manifestations led to the diagnosis of Blau syndrome. Complications of ocular involvement include cataract, glaucoma, and chorioretinitis. Therefore a close ophthalmologic followup is essential, [8].

Atypical cases are described with liver, renal, vascular, and pulmonary involvement [14, 15]; granulomatous pulmonary involvement seen in our case is a very rare entity, previously pulmonary involvement presenting as interstitial pneumonitis has been described by Becker et al. [15].

Pathological analysis of involved tissues shows nonnecrotizing and noncaseating granuloma of the biopsied tissue. Laboratory analysis may show normal to markedly elevated inflammatory markers: including erythrocyte sedimentation rate and c reactive protein [4].

As a result of varied clinical manifestation and rarity of the condition, there is no optimal treatment for Blau syndrome. Glucocorticoids are generally the first line of management. If the response to glucocorticoid therapy is not satisfactory or maintenance dose is high, greater than 10 mg/day, then additional immunosuppressive agent is added. Biologic therapies including tumor necrosis factor inhibitor and Interleukin 1 receptor antagonist have been used [10, 12]. Response to either is unpredictable, especially with regards to ocular improvement. Thalidomide suppresses nuclear factor kappa B activation, interferes with monocytes activation and proliferation, and has been shown to be effective in few cases.

## Key Message

Blau syndrome is a rare multisystem disorder which may be misdiagnosed as Juvenile Idiopathic Arthritis. Uveitis is the major cause of morbidity and should be intensively treated.
